# Beyond 50. challenges at work for older nurses and allied health workers in rural Australia: a thematic analysis of focus group discussions

**DOI:** 10.1186/1472-6963-11-42

**Published:** 2011-02-21

**Authors:** Lyn J Fragar, Julie C Depczynski

**Affiliations:** 1 Australian Centre for Agricultural Health and Safety, University of Sydney, 5 Greenbah Rd. Moree NSW 2400 Australia

## Abstract

**Background:**

The health workforce in Australia is ageing, particularly in rural areas, where this change will have the most immediate implications for health care delivery and workforce needs. In rural areas, the sustainability of health services will be dependent upon nurses and allied health workers being willing to work beyond middle age, yet the particular challenges for older health workers in rural Australia are not well known. The purpose of this research was to identify aspects of work that have become more difficult for rural health workers as they have become older; and the age-related changes and exacerbating factors that contribute to these difficulties. Findings will support efforts to make workplaces more 'user-friendly' for older health workers.

**Methods:**

Nurses and allied health workers aged 50 years and over were invited to attend one of six local workshops held in the Hunter New England region of NSW, Australia. This qualitative action research project used a focus group methodology and thematic content analysis to identify and interpret issues arising from workshop discussions.

**Results:**

Eighty older health workers from a range of disciplines attended the workshops. Tasks and aspects of work that have become more difficult for older health workers in hospital settings, include reading labels and administering medications; hearing patients and colleagues; manual handling; particular movements and postures; shift work; delivery of babies; patient exercises and suturing. In community settings, difficulties relate to vehicle use and home visiting. Significant issues across settings include ongoing education, work with computers and general fatigue. Wider personal challenges include coping with change, balancing work-life commitments, dealing with attachments and meeting goals and expectations. Work and age-related factors that exacerbate difficulties include vision and hearing deficits, increasing tiredness, more complex professional roles and a sense of not being valued in the context of greater perceived workload.

**Conclusions:**

Older health workers are managing a range of issues, on top of the general challenges of rural practice. Personal health, wellbeing and other realms of life appear to take on increasing importance for older health workers when faced with increasing difficulties at work. Solutions need to address difficulties at personal, workplace and system wide levels.

## Background

The rural health workforce in Australia is ageing and is older than the urban health workforce [1-4]. In 2005, the average age of nurses in outer regional areas of Australia was 46.0 years, compared to 44.6 years in metropolitan areas [2]. Although younger than the nursing profession, rural allied health workers also tend to be older than their city counterparts [3]. Both the nursing and allied health workforces are predominantly female, at around 75% [4].

For the Hunter New England Area Health Service (HNE Health), in northern New South Wales, around one quarter of allied health workers and a half of the rural nursing workforce were aged over 50 years in October 2009 (HNE Health Workforce Informatics). This a pattern likely to be shared by other rural health services across Australia. Workforce sustainability, at least in the short term, is likely to depend on nurses and allied health workers being willing, able and happy to work beyond middle age.

Ageing is associated with recognised physical and mental changes including reductions in aerobic power, muscular strength and endurance, reaction speed, acuity of special senses, difficulties with thermoregulation, sleep disturbances and concerns about increased risk of chronic illness [5-8]. Back pain, other musculo-skeletal disorders and stress-related mood disorders are common health and injury problems with older health workers [9,10]. However, in some studies, older nurses and other older workers have been shown to have better than average physical and mental health - although this may be because work helps keep older workers healthy, or less healthy older workers leave the workforce [9,11].

Generally speaking, in the absence of major illness, little relationship has been found between ageing and work performance per se [5,12-14]. Personal health and fitness however, can be highly variable amongst older adults and is more likely to affect physical strength and likelihood of injury than ageing itself [5,12]. Poor health is strongly associated with labour force exit [15-17]. Whilst older workers have considerable capacity to manage job demands and difficulties, it has been suggested that at some point, older workers do become "overwhelmed" by the increased risk of health consequences, injury and disability [18].

Recruitment and retention of health professionals of all ages has proved an ongoing difficulty in rural areas. This is related to an interplay of personal, environmental and wider work-related factors. Professional isolation, lack of recognition limited access to professional education, high workloads and stress related to lack of replacement staff, long work hours, limited resources and sometimes unsupportive management practices are commonly described workplace challenges [3,19-22].

Given the proportion of rural health workers aged over 50 years, the question to be addressed is whether there are specific difficulties for older workers on a day to day basis, in addition to the challenges faced by all health workers? Specifically, this study aimed to identify work tasks and broader challenges that have become more difficult for rural health workers as they have become older and the factors that contribute to these difficulties. Findings are informing the development of solutions at personal, workplace and system-wide levels, in an ongoing action research project.

## Methods

This qualitative research project used a focus group methodology and thematic analysis to identify and interpret issues arising from workshop discussions. Participants were also encouraged to reflect on issues, provide further input and act locally on ideas whilst the wider research was still in progress, consistent with qualitative action research principles.

A Project Reference Group (PRG) was established prior to commencement of the workshops, which consisted of the two researchers and eight health service personnel. These included health service managers, rural nurses and allied health workers currently working within HNE Health. PRG members were nominated by the HNE Health Executive Team on the basis of particular areas of practice, location, responsibility or expertise relevant to the Project. The purpose of the PRG was to provide practical advice and assistance on the conduct and direction of the project; provide feedback after the initial workshop; and to review and provide practical comment on preliminary results and recommendations.

**Figure 1:**
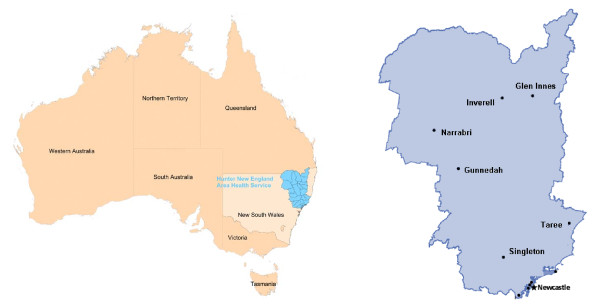
**Location of communities where older health workers project workshops were held within Hunter New England Area Health Service, NSW, Australia**.

Communities were selected to host a focus group or 'workshop' on the basis of size and geographic spread across the Hunter New England region of rural NSW. Each workshop was held in a town with a population between 5,000 and 30,000 people. Communities were located in six of the seven rural management clusters of HNE Health (Figure 1).

Local health service managers or their representatives were contacted to assist in arranging the workshop and to make sure all health service staff knew about it. Notification of each local workshop, with an invitation to participate directed at staff '50 years and over', was circulated 2-3 weeks in advance, through regular workplace communication channels. This included staff email networks, staff meetings, staff notice boards and word-of-mouth. Health service managers in communities within an hour's drive of the hosting health service were also asked to inform their staff about the workshop.

A convenience sample of 80 older health workers attended the six workshops between August and November 2008. Four were men and an average of 13 participants attended each workshop. Demographic information on the age and characteristics of individual participants was not collected, but each group was asked to indicate their general work experience and fields of practice. Consistent with an 'over 50' cohort, a number of participants had worked within the health service for 30 years or more.

Participants were mainly drawn from the nursing and allied health professional groups, who were the focus of recruitment efforts. Allied health fields of practice included radiography, occupational therapy, diversional therapy, physiotherapy, social work and psychology. Nursing sector participants included registered nurses and enrolled nurses from community health, aged care, general hospital wards, operating theatres, sterilising departments, midwifery, emergency departments and health service management. A small number were from other professional groupings (less than 10%), encompassing 'other professional, para-professional and clinical support staff' (Aboriginal health), 'corporate services' (clerical administration) and 'hotel services' (catering). The majority of participants worked regular office hours, although others had worked shift work in the recent past.

Participation was completely voluntary and informed consent was obtained from participants prior to their involvement. At the outset of each workshop, the purpose of the research was explained to participants by the lead researcher, who acted as the group facilitator. A 'participant information sheet' was provided outlining further details; and after opportunity for questions, participants were asked to sign a formal consent form prior to commencement of discussions.

Within each workshop, participants were asked to identify and describe:

• work tasks and aspects of work that have become more difficult for rural health workers as they have become older;

• age-related changes and other exacerbating factors contributing to these difficulties

Responses focusing on these questions were recorded by participants during small break-out groups of 3-5 people. In particular, participants were asked to list difficulties in a table; and to record ideas on the age-related changes and exacerbating factors that contributed to each of these difficulties in an adjacent column. One or two items from each small group were selected for further discussion by the larger group, enabling other group members to contribute and/or debate ideas. One researcher had the designated task of taking detailed notes during the course of these larger group discussions, which supplemented the data recorded by participants on the small group worksheets.

Thematic content analysis was then used to identify issues arising from workshop discussions. Notes and worksheets were reviewed after each workshop and information sorted into categories that focused on defining difficulties associated with specific tasks, variation within work contexts and relationships between categories. Categories were progressively reviewed and used to help guide discussion in subsequent workshops until no new information on the subject arose. Themes relating to difficulties were tabulated moving from (1) particular tasks and specific workplace settings, to (2) more general aspects of work and broader settings.

Participants were invited to reflect on ideas shared in their 'workshop' and act locally on issues whilst the wider research project was still in progress. Preliminary findings from all workshops were sent to participants, who were given opportunity to provide further feedback and ideas prior to final recommendations being made.

The research protocol was approved by the Hunter New England Human Research Ethics Committee (Reference 08/05/21/4.06). This was consistent with local health service requirements and research ethics principles of the World Medical Association Declaration Of Helsinki [23]

## Results

### Challenges in particular work settings

The following tables indicate specific tasks and challenges in a range of work settings that older rural health workers reported as becoming more difficult with age. Work and age-related factors that contributed to these challenges, are also presented.

**Table 1:** Tasks and challenges in hospital ward settings that have become more difficult with age

Tasks impacted by age-related factors	Reported reasons why each is more difficult
**1. Reading drug labels and information sheets**	*Age related factors:*
**2. Reading other print communications**	▪ Deteriorating vision
	*Exacerbated by:*
	▪ Poor light at night, environmentally friendly light bulbs
	▪ Small print (eg. drug labels, information sheets, ampoules, imprints on foil packs)
	▪ Colour of print (eg. orange or red writing on ampoules)
	▪ Reading through plastic sleeves
	▪ Losing glasses - continually taking them on and off
	▪ Increased computer work - associated eye strain
	▪ Size of phones, keypads, text messages

**3. Administering medications**	*Age-related factors specific to these tasks -*
Including:	▪ Reduced strength in hands and wrists
▪ Cracking ampoules	▪ Pain in hands and wrists
▪ Administering IV medications and removing IV lines	▪ Fine motor co-ordination reduced
▪ Openning packages - lids, jars, plastic overwrap, boxes with tape, dressing packs, seals, child proof packages, IV fluid bags	▪ Reduced dexterity
	▪ Increased cramping of fingers
	*Exacerbated by:*
	▪ Smaller, more secure packaging
	▪ Environmentally friendly gloves

**4. Hearing in the hospital ward or unit**	*Age related factors:*
*In particular:*	▪ Deteriorating hearing
▪ Hearing patients	▪ For some, noise induced hearing loss
▪ Hearing at the work- station	
- Drug orders, other instructions and conversation over the phone	*Exacerbated by:*
- Other staff	▪ Accents of some non-English-speaking staff
- Alarms and distinguishing between them	▪ High background noise level of wards with open office
	▪ Distractions - constancy of phones ringing, others talking
	▪ Anywhere where there's a crowd
	▪ Speech of younger staff

**5. Manual handling - lifting and/or moving patients and equipment**	*Age-related factors:*
*In particular:*	▪ Musculo-skeletal changes affecting strength, muscle tone, flexibility
▪ Examining patients	▪ Stability and balance
▪ Dressing patients - shoes & socks, adjusting clothes/attire	▪ Increased pain, stiffness (+/- osteoarthritis) in:
▪ Holding limbs and draping surgical patients	- Joints - hips, knees, hands, feet
▪ Pushing/pulling equipment - eg. beds, chairs	- Neck and shoulders
▪ Showering patients	- Back
▪ Toileting patients in difficult areas	▪ Manoeuvring more difficult when older; fuller figures of both patients and staff
▪ Squatting or kneeling - for procedures, picking things up off floor	*Exacerbated by:*
▪ Doing dressings	▪ Manoeuvrability and maintenance of equipment
▪ Making beds, adjusting bed heights	▪ Workplace ergonomics and design of facilities (old)
▪ Walking up and down steps	▪ Narrow bathrooms and doors don't allow room for lifting aids
▪ Transporting objects, records	▪ No shelves or poor position of shelves
	▪ Unco-operative patients
	▪ All-in-one gowns - difficult for examining patients

**6. Shift work**	*Age-related factors:*
*In particular:*	▪ Tiredness, especially after lunch
▪ 10 hr shifts, longer shifts, more shifts, double shifts, early shifts, split shift	▪ Reduced stamina from physical demands on body
▪ Rigid roster	▪ Longer recovery periods - "takes 2 days to get over a double shift"
▪ On call	▪ Lack of sleep, disturbed sleep patterns, "waking at 3 am"
▪ Long working days	▪ More anxious, not dealing with lack of sleep as well as before
	▪ Strong work ethic of older workers - "if you were younger, you would just go off"
	*Exacerbated by:*
	▪ Inflexible work hours
	▪ Lack of staff - "can't go off sick, no one to replace you"
	▪ Unable to take time out to recover
	▪ Poor recovery after inconsistent shifts; insufficient rest times between rotations and being on call
	▪ Some older workers more resistant to shift changes

**7. Long periods of standing, walking or sitting**	*Age-related factors:*
*In particular:*	▪ Manoeuvring more difficult when older, fuller figures of both patients and staff
▪ Sitting down for long periods eg. data entry	▪ More difficult to get mobile quickly after sitting, due to stiffness and back problems
▪ Standing/walking, being on your feet for long periods or all day. eg. in operating theatre	
▪ Unnecessary walking	*Exacerbated by:*
	▪ Past surgical procedures
	▪ Design of facilities - long distances to medication rooms, utility rooms
	▪ Running phones up and down to patients, "have to leave what you are doing"

**8. Midwifery**	*Age-related factors:*
*In particular:*	▪ More difficult to lean, bend, stand for long periods now older
▪ Delivery of babies - long periods of:	▪ Back pain and stiffness
- Leaning over beds	▪ *(As above for manual handling)*
- Bending	
- Being on your feet	*Exacerbated by:*
- On floor with mother	▪ New options/positions for birthing & birthing chairs
	▪ Presentations now more complex, with more requirements

**9. Physiotherapy**	*Age-related factors:*
*In particular:*	▪ Back pain and stiffness
▪ Patient exercises - bending/reaching over beds leading to back-strain	▪ *(As above for manual handling)*
	*Exacerbated by:*
	▪ Allied health workers in rural areas are often sole practitioners with no help

Work in the hospital setting that has become more difficult for older workers includes reading medication labels. This is exacerbated by poor lighting, small print size and print colours, such as orange-red print on ampoules used for injections. Hearing difficulties are exacerbated by high levels of workplace noise and for some, by the accents of staff from non-English-speaking backgrounds.

Manual handling and manoeuvring of patients was particularly difficult where squatting, bending or maintaining postures for long periods was required. It was also considered more physically challenging, because patients were getting older, more debilitated and overweight.

"Patients are older and heavier ... and so are we."

Shift work was met with increased tiredness with an expressed need for longer recovery times between shifts. Disturbed sleep and anxiety about lack of sleep was commonly reported, with problems of tiredness sometimes exacerbated by a lack of replacement staff.

"It takes 2 days to get over a double shift."

"You can't go off sick - there's no one to replace you."

Specific clinical procedures, such as physiotherapy exercises, are also more difficult because they often involve long periods of leaning, bending and being on one's feet. Midwifery had become more of a challenge for older midwives, partly attributed due to the increased birthing options and positions for women.

"I think (these are) reasons why some older nurses no longer practice midwifery."

Challenges facing older community health workers are described in Table 2:

**Table 2:** Tasks and challenges in community settings that have become more difficult with age

Tasks impacted by age-related factors	Reported reasons why each is more difficult
**1. Work involving vehicles**	*Age-related factors specific to these tasks -*
*In particular:*	▪ Arthritis, sore joints, backaches - caused or exacerbated by vehicles and driving
▪ General driving during the day	- Less flexibility, manoeuvrability for getting in and out of cars
▪ Reversing vehicles	- Harder to turn around to look behind/harder to see out of cars
▪ Getting in and out of cars and the '*Day Care' *bus	▪ More fatigued for report writing, after driving around during the day
▪ Carrying equipment to/from vehicles and homes eg. baby scales	*Exacerbated by:*
	▪ Ergonomics of smaller and lower cars ("green policy")
	- Hard to get in and out of, not comfortable, poor seating
	▪ '*Day Care*' bus, bumping up and down along the road
	▪ Tight time frames, rushing
	▪ General safety issues related to night and long-distance driving - vision, fatigue, time allowed

**2. Work involving home visits**	*Age-related factors specific to these tasks -*
*In particular:*	▪ Musculoskeletal changes - strength, tone, flexibility, pain and stiffness, backache - caused/exacerbated by vehicles and driving
▪ Carrying equipment to/from vehicles and homes eg. baby scales	▪ Manoeuvring, mobility and stability on feet (with clutter) more difficult when older
▪ Moving around and manoeuvring in cluttered houses	*Exacerbated by:*
	▪ Home clutter and trip hazards
	▪ Manoeuvrability of equipment
	▪ Home ergonomics and design - narrow bathrooms and doors that don't allow room for manoeuvring

Community health workers described greater driver discomfort and fatigue now they are older. Getting in and out of vehicles was more difficult - especially with the purchase of smaller, lower cars. Carrying equipment to and from vehicles and moving around in cluttered homes, has made home visiting more difficult.

"The (smaller car brands) are too low - you have to 'roll out of them.'"

Work with computers (Table 3) also poses a particular challenge for older health workers. The problems relate to physical discomfort associated with sitting for long periods, keyboard dexterity and seeing small font on screens. Additionally, there are mental challenges associated with the rapid adoption of new programs, the need to remember passwords and interpretation of computerised results. Little previous exposure and a general lack of confidence with computers was a commonly reported experience. It was also felt group training sessions were mostly levelled at a younger, more computer literate generation.

**Table 3:** Challenges associated with computer work that have become more difficult with age

Challenges impacted by age-related factors	Reported reasons why each is more difficult
***1. Physical aspects***	*Age-related factors*
*In particular*	▪ Neck, shoulders strain, tired at end of day
▪ Use of mouse, developing carpel tunnel	▪ Stiffness when sitting for long periods
▪ Keyboard dexterity	▪ Hand problems, clicking on mouse
▪ Seeing screens	▪ Eye strain and vision
▪ Sitting at computers for long periods for data entry	*Exacerbated by:*
**2. *Mental aspects***	▪ More clicking on the mouse required for newer programs
*In particular:*	▪ Ergonomic factors:
▪ Use of new technology	- Sitting more, less active
▪ Learning new programs and changes to programs	- Posture and seating, some computers badly set up, on a bench
▪ Statistics	▪ IT education provision
▪ Remembering passwords	- not tailored to particular needs
▪ Interpreting computerised results	- Not enough time allowed for older workers to learn, absorb and apply knowledge. "Younger workers already with computer skills, get the same amount of training time"
	- "Helpdesk" is on the computer, not in hardcopy"
	▪ Historical-generational learning factors
	- Not having computers at home and not growing up with computers
	- Computers not seen as a priority
	- Learn differently, "we are more practical people"
	- Harder for shift workers to adapt to computer
	▪ General lack of confidence and support with computers and new technology:
	- IT support not always available when needed
	- Frequency of use - affects confidence
	- Tendency to avoid new technology due to difficulty with equipment

"I.T trainers assume a skill level that is not necessarily so"

"Some have even left because of the computers... or some can't work in emergency department (ED) ... because they can't use the computer."

Participation in meetings, conferences and education programs often requires long distance travel and night driving (Table 4). This has become harder now that participants are older, due to driver fatigue, musculoskeletal problems and difficulties with night vision. These issues are exacerbated by a lack of adequate back support in vehicles, glare of lights and bright road signs. Alternatives such as teleconferencing also pose problems for the hearing impaired.

**Table 4:** Challenges associated with meetings and ongoing education programs that have become more difficult with age

Challenges impacted by age-related factors	Reported reasons why each is more difficult
**1. Travel to meetings and conferences**	*Age-related factors:*
*In particular:*	▪ Less stamina, staying power for long distance driving
▪ Long distance and night driving to meetings	▪ Night driving:
	- Glare of lights, vision not as sharp
	- "Lots of signs, adds to confusion"
	*Exacerbated by:*
	▪ Ergonomics of smaller and lower cars
	▪ Tight time frames (rushing)

**2. Participation in ongoing education**	*Age related factors:*
*In particular:*	▪ Difficulty retaining 'new learnings'
▪ Going to educational courses	*Exacerbated by:*
▪ Difficulty learning new skills eg. surgical procedures	▪ Can't find time to go, can't find the time to read about courses
▪ Negative responses to older workers wanting/participating in training	▪ Locations - hard to get to (see travel difficulties)
	- Usually located hours away - time to get there and back
	- Very early leaving and very late getting back
	▪ Attitudes toward older workers showing an interest or participating in courses:
	- Offered to younger staff in preference
	- Feeling of being overlooked for courses, "you'll be retiring soon" ...but a prevailing belief it is more likely older workers will stay in a place for longer
	▪ In contrast, participants also reported that some workers "flatly refuse to go/change or learn - doggedness" (which doesn't help those who want to)

**3. Hearing speech in video and teleconferencing meetings**	*Age related factors:*
	▪ Deteriorating hearing - 'noise induced' for some
	*Exacerbated by:*
	▪ Nature of communication system

" Once upon a time I could get in a car and drive to a meeting at the end of the day. Now I can't do that."

### Challenges spanning work tasks and settings

Table 5 indicates the more general challenges associated with age and work within rural health services. These difficulties span across work settings and particular tasks.

**Table 5:** Challenges reported that span work settings and particular tasks

Challenges impacted by age-related factors	Reported reasons why each is more difficult
**1. Dealing with mental demands of work**	*Age-related factors:*
*In particular:*	▪ Memory and recall not as good as when younger
▪ Dealing with the paperwork	▪ Added stresses affect cognitive function
▪ Remembering things	▪ More difficult to concentrate for long periods and with distractions
- Passwords	▪ Mental fatigue leads to tiredness
- Clients names	*Exacerbated by:*
- Where "lists are"	▪ Constant changes in work systems
▪ Emails and other required reading	▪ More hats to wear - roles, variety of tasks and multi-tasking
▪ Keeping mind on task	▪ Information overload
▪ Managing stress and burn-out	- Increased emails, required reading, amount of reading within tasks
	- "Too many meetings"
	▪ Stress and expectation to have "stuff in your head"
	- New information and passwords *(see computers)*
	- Client expectations to remember their names
	▪ Pressure of meeting several demands at once
	▪ Longer term impacts - always stretched, physical and emotional stress, less happy, less tolerant etc.

**2. Meeting the physical demands of workload as an older health worker**	*Age-related factors:*
	▪ Musculo-skeletal changes - increased pain, stiffness
	▪ General tiredness and fatigue - especially after lunch, end of day, focusing for long periods
	▪ Reduced fitness and endurance - reduced energy levels and loss of weight (or gain)
	▪ Slower physically - takes longer to do things, including routine tasks, can't walk as quickly
	*Exacerbated by:*
	▪ A sense there is more to do, but not enough time to do everything, or do it well
	▪ "Patients (and populous) are older, heavier- and so are we"
	▪ Sicker, more dependent and debilitated patients

**3. Coping with change**	*Age related factors:*
*These may be:*	▪ Subdued mental reflexes/agility
▪ Procedural	▪ Need more time to assimilate knowledge/change
▪ Organisational	▪ Less/lack of adaptability
*In particular:*	
▪ Role changes	*Exacerbated by/resistance to change due to:*
▪ Coping with change both cognitively and emotionally	▪ "Constant change" referring to the many changes in the health service over the past 15 years eg. in systems, procedures, roles
Coping with sudden change	▪ Change often associated with increased paperwork requirements, new computer programmes
	▪ Less tolerance for supposedly 'new things' and "reinventing wheels"
	▪ Experience of older workers not valued, "people don't ask us (about the change), the opportunity is not provided"
	▪ Casual workers - don't always know about changes
	▪ In contrast, participants also noted that some workers refuse to change or learn - "doggedness")

**4. Dealing with emotional impacts of attachment, loss and awareness of own ageing and mortality**	*Age related factors:*
*In particular:*	▪ Confronted by ageing process in clients, colleagues
▪ Emotional stress after episodes in acute care	▪ Death of older people affects us more as we age - closer to own age"
▪ Death of older people, who are often well-known to workers	▪ Dealing with ageing in clients and own ageing/mortality
▪ Loss, grief associated with colleagues leaving	▪ Slower recovery from physical and emotional stress
Coming to work with younger colleagues	▪ Difficulty sleeping
	*Exacerbated by:*
	▪ Patients are becoming peers, bonds formed over any years - "Now you know them, they're not just a patient"
	▪ No debriefing available

**5. Dealing with emotional impacts of not achieving personal standards and goals**	*Age related factors:*
Including:	▪ Self-awareness that things take longer/workload
▪ A sense of change from total patient care to something less, with lower standards	*Exacerbated by:*
▪ Lack of time to achieve higher standards of care	▪ Difficulty saying no to physical work required (ethic)
▪ Less job satisfaction:	▪ Lack of time to achieve high standards leading to feelings of 'guilt, frustration, lack of pride, poor job satisfaction'
	▪ Seeming lack of understanding by higher executives of encroachment of administrative work on clinical time
	▪ Declining work conditions over the years - shared offices, technology, work cultural changes, more stimulation

**6. Emotional impacts of not meeting/fulfilling expectations of others**	*Age related factors:*
*Including:*	▪ Less able to cope with stress and changing roles (*see above)*
▪ Patients expectations	*Exacerbated by:*
▪ Organisational expectations relating to workload and workforce:	▪ Nature of job - increasingly stressful and changing roles at a time of life when less able to cope
▪ Peer expectations - supporting other staff and being the "stable" staff member	▪ High expectations of clients for complete recovery and short recovery times mismatched with staff perspective - leading to tension/stress
	▪ High organisational expectations relating to workload
	- Sense that required documentation is largely driven by fear/risk of litigation
	- Workforce issues - recruitment in rural areas, getting harder
	▪ Supporting other staff - exhausting
	- "Others revolve, rotate around you and you have to take on more of the support/responsibility role"

**7. Balancing work/family life commitments**	*Age -related factors:*
*Including:*	▪ Own health issues - need for health appointments, specialists
▪ Juggling work, family and sharing time with all family members	▪ Coping with the many facets of life and still keeping your mind on the job
▪ Time to fulfil all commitments	▪ Less energy, slower, more weary, yet seemingly more tasks to do
▪ No time for self - to look after yourself	▪ Wisdom - desire for balance of work and family life
	*Generational factors:*
	▪ Main carer role in family -
	- Ageing parents
	- Looking after husband, older parents, growing children, adult children needing help eg. minding grandchildren
	- Going home to do more physical work
	*Exacerbated by:*
	▪ Other shift workers in family
	▪ Split days off
	▪ Distances to travel to see family
	▪ "More to do (family), less time to do it",
	▪ Assumptions by organisation and younger colleagues that older workers don't have family commitments

**8. Staying engaged and positive in spite of difficulties**	*Age related factors:*
*Including:*	▪ Less capable of dealing with the increased physical/emotional workload posed by demanding patients (*see above*)
▪ Maintaining tolerance for attitudes and behaviours of patients, organisation **and **younger workers	- On the one hand, tolerance often lower for rudeness of people - "get to an age where you say - hang on, you can't speak to me like that"
▪ Maintaining tolerance for attitudes and behaviours of patients, organisation **and **younger workers	▪ In contrast: wisdom and tolerance gained through experience
	- "As you get older, you realise you can't fix things... you can only do so much"
	▪ Sensitivities and the "psychology of ageism"
	- More sensitive to criticism when older
	- Negative thinking/spin from others and from self eg. "I'm too old"
	▪ Tiredness dealing with difficult situations eg. confrontation associated with "dealing with destructive and undermining behaviours - would rather avoid it"
	*Exacerbated by:-*
	▪ Attitudes and behaviours of patients, organisation, younger workers
	▪ Interpersonal communication
	- Communication with younger workers difficult - language used, seem "less compassionate", seem to "only half listen"
	- In contrast: "Not in our team, we work well and communicate well," (need to) "beware of generalising"

Participants felt that meeting overall workplace demands across settings had generally become more difficult, as workers have become older. Mental fatigue is occurring from a sense of 'information overload' and an expectation to have 'stuff in your head'. Increased paperwork requirements and having multiple roles within the health service, are contributing to a build up of mental stress, making it difficult to concentrate amidst distractions and leading to a sense of 'always feeling stretched'.

"It's just harder when things mount up at end of the day."

Personal challenges reported by older workers include coping with 'change,' such as increased role complexity, 'more hats to wear' and greater administrative requirements.

"A new system every year, you learn it, then it changes again."

This is reportedly worse when older workers 'organisational wisdom' about change and what has worked in the past, has not been sought or valued. 'New ideas' were sometimes perceived as 'reinventions' that led to more work, more stress and little perceived benefit.

"What goes around comes around, a revolving door of ideas, ... you've done it before and nothing ever came of it."

Emotional stress following acute care episodes or death of people, has also become much harder to cope with, with workers often knowing people personally, after years of caring for them. Participants also reported a greater sense of empathy, attachment, loss and an increased awareness of their own ageing and mortality.

"The death of older people affects us more as we age ourselves - they're closer to our own age now."

"Now you know them, they're not just a patient."

Balancing work with family life commitments is another challenge. Many described having an extended carer role within families - for ageing parents, partners, grown up children and grand-children. This could involve travelling long distances for health and family matters. Participants expressed a desire to work less or more regular hours, or have time off to attend to these responsibilities. However, they felt management often did not understand this need, or that there were not enough workers available in small rural health services to enable this.

"When finished work, (we are) still carrying the bricks - caring for our own sick elderly."

"At our age, we have children growing up, with their children, and elderly parents, (we are) the main carer."

Participants were keen to make a positive contribution to good health care in their community - and believe they were doing so. However, at the same time, many felt they fall short of achieving personal standards related to care - or in fulfilling the expectations of patients (related to care), the organisation (relating to workload) and peers (related to support). This was attributed to high workloads, reduced clinical time, increased administrative tasks and a sense they could not get done in a day what they wanted or were expected to do. Participants felt this was worse for older workers, because they were slower than they used to be, yet had a high work ethic and pride in delivering quality care.

"It's not the work you do, it's the work you cannot do that is frustrating and emotionally distressing and results in less job satisfaction."

Given this context, some expressed a lowered tolerance for uncooperative, rude or aggressive patients, or those who 'won't help themselves'. Conversely, some felt that life experience gave them a more 'empathetic' view than when they were younger.

Participants in each workshop were offered the opportunity to suggest potential solutions to the problems that were identified. These solutions fell into three categories - recommendations for personal behavioural changes, for work-supportive solutions (by local and area health services) and system-wide solutions such as purchasing of vials with more legible print. These ideas are being incorporated into an ongoing action-research project by the health service.

## Discussion

This study attempted to capture a range of perspectives on this issue from older health workers in rural health services across the Hunter New England region, although it could not be claimed that the views of participants were 'representative' of all older health workers in the area. Recruitment of individuals was dependent upon participants receiving information about the workshops; the appeal of attending a 'workshop' on this issue; and available time on the part of managers to encourage local participation and ensure information was received by all health workers. Whilst provisions were made for work release to attend workshops, fewer 'current' shift workers attended workshops than community health staff and others who worked more regular hours. Also, whilst groups were facilitated, it is possible some individuals did not feel free to express personal opinions in a group forum; and that some individual opinions were not captured by group data collection processes.

Despite these study limitations, recurrent themes arose in the workshops relating to broad areas of difficulty, with no new themes on the issue emerging by the last workshop. Ageing changes described by older health workers that contributed to difficulties at work, are generally consistent with ageing impacts reported elsewhere [5-8]. In particular, musculoskeletal problems, deteriorating vision, fatigue and reduced fine motor dexterity were common concerns.

Participants were concerned that these difficulties were affecting their sense of personal health and well-being. A desire for reduced work hours and to spend more time with family, suggested an awareness of the impacts of 'too much work', supporting findings elsewhere that the benefits of a 'healthy worker effect' can be compromised by 'work-family' conflict and excessive work hours [9,11,24] Physical demands, rotating shifts, inflexible scheduling and full time positions, are also known to be associated with chronic fatigue and personal health concerns of older nurses elsewhere [6,9,25]. However, alternatives such as lateral movement of older workers to less physically demanding positions cited elsewhere [10,26,27], are a less likely option for many small rural health services, already short of staff.

Difficulties working with computers were commonly reported, supporting findings elsewhere [28,29], including the sense that computerised systems did not necessarily reduce documentation time [30]. Participants were keen to participate in computer education courses, given attention to more appropriate, adult learning styles - a finding also supported by other studies [5,12]. However, barriers to education access for older workers not reported elsewhere, include difficulties with night and long distance driving and difficulties hearing teleconferencing facilities.

Whilst a sense of community and appreciation of rural lifestyle are amongst the most important reasons given by health workers, for continuing to work in rural areas [19,31,32], there is also a greater likelihood that patients in rural areas are known by health workers, with emotional implications when well-known patients suffer or die. The grief and emotional impact described by participants of 'losing someone you know' may be a form of 'compassion fatigue', also described as the 'cost of caring for others in emotional pain' [33,34]. Given participants' life stage and the rural context, 'compassion fatigue' may be part of the fatigue experience of older rural health workers.

Reviewing life-work balance has been reported as a response to working conditions, existence of chronic pain, ageing and personal issues [10]. Prominent among 'personal' issues in this study and elsewhere [6,35], are the demands and complex responsibilities associated with an extended primary carer role outside of work - contributing to a desire to work less hours.

Similar to other studies [10,36], participants felt that the perspectives of older workers were often not valued by the organisation, even though worker participants were generally supportive of the organisation and happy to have been consulted in this study. A sense of powerlessness about not having one's opinion heard, has been associated with burnout and ill-health in health workers [10]. More generally, work stress amongst rural nurses has been attributed to a climate of change, restructuring and financial constraints that lead to increased expectations or role changes for which nurses are not prepared or consulted [37]. This experience may be worse for older workers, also dealing with ageing and generational factors that make adapting to change harder.

As a generation, older health workers have been described as committed, hard-working, intuitive and skilled decision makers, possessing a deep-rooted understanding of patient needs [6,26,38]. However, such values may increase the emotional exhaustion and guilt felt when extra stressors related to role change and patient load, takes time away from providing the quality care that helps define self-worth [6,39,40].

Intolerance for perceived rudeness, abuse and unwillingness in some patients to 'help themselves', may arise because such behaviours conflict with generational values; and because older health workers feel they are already providing quality care under stressful circumstances. For example, obese patients were regarded as more physically demanding, which, if combined with a perception that a patient was not trying to help themselves, could contribute to or explain some of the negative attitudes toward such patients, that have been reported elsewhere [41]. However, in contrast, other participants felt they were more empathetic or tolerant of 'demanding' patients than younger workers - a finding also reported elsewhere [41,42].

Generally speaking, older health workers displayed a great sense of humour during the workshops and believed their maturity and wisdom gave them a valuable perspective on work and life. Like older workers elsewhere, participants 'continue to care', despite difficulties, intergenerational conflicts and less respect from patients [10,39].

Collegial relationships within the health care team have been found to reduce job stress and foster decisions to stay in the workplace [6], whilst poor working conditions, lack of supportive workplace relationships, combined with ageing concerns, have influenced older nurses to make changes for the sake of their own health [10]. Finding ways to address concerns and avoid excessive demands being placed on older health workers, should contribute to a happier, healthier and more sustainable older health workforce.

## Conclusions

This study describes some of the practical difficulties and issues confronting older workers in the Hunter New England health region of NSW, which are likely to be shared in other regions. With a view to developing practical solutions, the following recommendations have been made to and accepted by the Health Service Executive Team:

- Older health workers should be involved in development of "*a resource booklet" *on ageing and other factors that impact upon work with practical suggestions for addressing these at personal and local level

- The Health Service should establish a health service "*Task Force"*, comprising managers, older rural health workers and an occupational therapist, to examine the study findings and implement area-wide policy and practice solutions, as well as recommendations for state-wide policy development.

Older health workers in rural areas are a committed and productive section of the workforce who are meeting health service delivery needs at considerable personal cost. This study supports the view that there comes a point where physical and emotional 'costs' exceed the benefits of work, particularly as other realms of life take on more importance - but that this is likely to occur more rapidly, where stressors and difficulties that are amenable to solution or modification are not addressed. Actions that health services can take to consult and value the opinions of older workers in addressing these difficulties, will benefit not only older workers, but will be in the interests of the health service and better health care delivery.

## Competing interests

The authors declare that they have no competing interests.

## Authors' contributions

Both authors have (1) made substantial contributions to conception and design, acquisition of data, analysis and interpretation of data; 2) were involved in drafting the manuscript; and 3) have given final approval of the final manuscript.

## Funding

The Older Health Workers Project was an internally funded, co-joint activity of the Australian Centre for Agricultural Health and Safety and the Hunter New England Area Health Service. No special-purpose or external funds were received or allocated from any other funding bodies. Research time and labour costs were met under existing funding arrangements by the Australian Centre for Agricultural Health and Safety.

## Pre-publication history

The pre-publication history for this paper can be accessed here:

http://www.biomedcentral.com/1472-6963/11/42/prepub
